# Supplementary material for: Gαi-derived peptide binds the µ-opioid receptor
Source: Pharmacol Rep. 2023 Feb 25;75(2):465–73. doi: 10.1007/s43440-023-00457-5 (PMC10060287; doi:10.1007/s43440-023-00457-5)
Supplement: Supplementary file 1 — Supplementary file1 (DOCX 39 KB) [file 43440_2023_457_MOESM1_ESM.docx]

**Gα_i_-derived peptide modulates binding of different ligands to the µ-opioid receptor**

Aleksandra Misicka-Kęsik, Jolanta Dyniewicz, Piotr Kosson, Piotr FJ Lipiński, Andrzej J. Bojarski, Stefan Mordalski

**Peptide synthesis**

The G-peptide was synthesized manually via Fmoc (9-fluorenyl-methoxycarbonyl) SPPS methodology with using Rink Amide resin and TBTU/HOBt/DIPEA as coupling reagents as was previously described by Niescioruk et. al. ^1^ Synthesis were carried out on Rink amide resin (0.5 g; 0.71 mmol/g, 200-400 mesh). Coupling reactions were conducted with 2 eq of amino acid (Fmoc-Phe-OH, Fmoc-Leu-OH, Fmoc-Gly-OH, Fmoc-Cys-OH, Fmoc-Asp-OH, Fmoc-Lys-OH, Fmoc-Leu-OH, Fmoc-Asn-OH, Fmoc-Asn-OH, Fmoc-Lys-OH, Fmoc-Ile-OH, Fmoc-Ile-OH), 2 eq HOBt*H_2_O, 2 eq TBTU and 4 eq DIPEA in DMF for 2h. The efficiency of coupling and deprotection reaction of each amino acids were monitored by Kaiser test. Fmoc groups were removed by using 20% (v/v) piperidine in DMF. After each deprotection step, the resin was washed 3xDMF (5ml). At the end the final amino acid was deprotected, peptide resin was washed 1x DMF (5 ml) and 3xMeOH (5ml) and dried under reduced pressure. The peptide was cleaved from the peptidyl-resin by 6 ml of mixture TFA/H2O/TIS (95%/2.5%/2.5%, v:v) for 3h. Mixture of TFA was removed under reduced pressure and peptide was participated by cold Et_2_O. Crude product was filtered and dried under reduced pressure. Purification of peptide was carried out by preparative RP-HPLC (Shimadzu, SHIM-POL A.M. Borzymowski, Izabelin, Poland) with Phenomenex Jupiter® 10 µm Proteo 90 Å, C_12_ Column (250 x 21.2 mm), AXIA™, and monitored at 210 (UV-Vis detector SPD-20 A) using a linear gradient 20 to 35% of B in 35 minutes (A: 0.1% TFA in H2O, B: 0.1% TFA in ACN, flow rate 20 ml/min). After purification we obtained 104,8 mg (76.1 mmol) of pure peptide in 15.9 % yield.


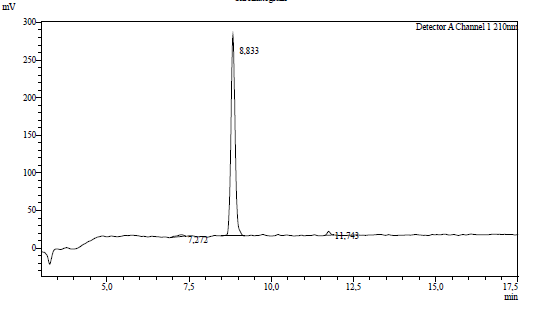
Characterization of G-peptide was done with a Shimadzu liquid chromatograph (LC)/MS system (Shimadzu, Kyoto, Japan) consisting of a Jupiter 4 µm Proteo 90 Å C_12_ column (250 x 4.6 mm, 4 um; Phenomenex, USA) a binary prominence pumps (LC-20AD), an autosampler (SIL-20ACHT), a solvent degasser (DGU-20A3R), a column oven (CTO-20AC), UV-Vis detector (SPD-20A), a controller (CBM-20A), a liquid chromatograph mass spectrometer with electrospray ionization (LCMS-2020). Analysis were performed with using a linear gradient 10 to 30% B in 10 minutes (A: 0.05 % FA in H2O, B: 0.05% FA in ACN, LC flow rate 1.2 ml/min, MS flow rate 0.4 ml/min). Detection was performed at 210 nm. ESI-MS (positive mode) = 459.85 [M+3H]^3+^ (calcd. m/z: 459.591), 473.50 [M+ACN+3H]^3+^ (calcd. m/z: 472.595), 689.30 [M+2H]^2+^ (calcd. m/z: 688,882), 1376.70 [M+H]^+^ (calcd. m/z:1376.75),(rt=8.388 min., purity 97.07 %). The chromatogram for pure peptide is shown at Figure 1.

Figure 1. The RP-HPLC chromatogram of purified G- peptide.

b)
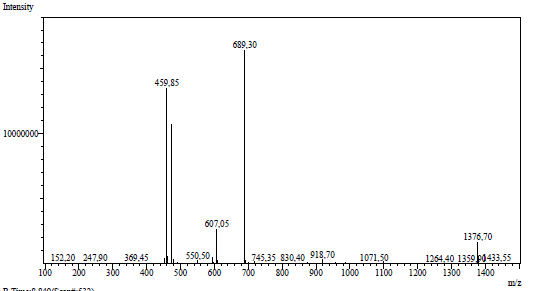


Figure 2. MS spectrum of the G-peptide with rt = 8.83 min.

(1) Niescioruk, A.; Nieciecka, D.; Puszko, A. K.; Królikowska, A.; Kosson, P.; Perret, G. Y.; Krysinski, P.; Misicka, A. Physicochemical Properties and in Vitro Cytotoxicity of Iron Oxide-Based Nanoparticles Modified with Antiangiogenic and Antitumor Peptide A7R. *J. Nanoparticle Res.* **2017**, *19* (5), 160.
